# Impact of acute and chronic exposure to sulfamethoxazole on the kinetics and microbial structure of an activated sludge community

**DOI:** 10.3389/frabi.2024.1335654

**Published:** 2024-04-02

**Authors:** Ilke Pala-Ozkok, Tugce Katipoglu-Yazan, Tugba Olmez-Hanci, Daniel Jonas, Emine Ubay-Cokgor, Derin Orhon

**Affiliations:** ^1^ Department of Chemistry, Bioscience and Environmental Engineering, Faculty of Science and Technology, University of Stavanger, Stavanger, Norway; ^2^ Environmental Engineering Department, Faculty of Civil Engineering, Istanbul Technical University, Istanbul, Türkiye; ^3^ Institute for Infection Prevention and Hospital Epidemiology, University Medical Center Freiburg, Freiburg, Germany; ^4^ The Science Academy, Istanbul, Türkiye

**Keywords:** acute inhibition, activated sludge, antibiotic resistance genes, modeling, chronic exposure, 454-pyro-sequencing, sulfamethoxazole

## Abstract

The aim of this study was to reveal the microbial and kinetic impacts of acute and chronic exposure to one of the frequently administered antibiotics, i.e., sulfamethoxazole, on an activated sludge biomass. Respirometric analysis and model evaluation of the oxygen utilization rate profiles were the backbone of this study. The results showed that continuous exposure to sulfamethoxazole resulted in the inhibition of substrate storage and an increase in the endogenous decay rates by twofold, which was supported by analysis of the resistance genes. A mild inhibition on the growth and hydrolysis kinetics was also observed. Moreover, sulfamethoxazole had a binding impact with available organic carbon, resulting in a slightly less oxygen consumption. DNA sequencing and antibiotic resistance gene analyses showed that continuous exposure to sulfamethoxazole caused a change in the community structure at the species level. Resistant bacteria including *Arthrobacter* sp. and members of the Chitinophagaceae and Intrasporangiaceae families were found to have dominated the bacterial community. The impact of intermittent exposure was also investigated, and the results indicated a drop in the severity of the impact after 20 days of intermittence.

## 1 Introduction

The contamination of water bodies with pharmaceuticals has been an issue for decades. Due to technical advances in analytical methods, many compounds can now be detected down to the nanogram per liter level. However, sulfamethoxazole (SMX) has a special place among the persistent organics present in water bodies because it has been used as human and veterinary medicine since 1969 and has been suggested to be persistent since 1985 (Straub, 2016). Due to its frequent use, bacterial resistance against SMX has developed; therefore, at present, it is administered in combination with trimethoprim (Drillia et al., 2005) to treat infections such as middle ear and urinary tract infections, bronchitis, and bacillary dysentery.

SMX belongs to the sulfonamide group of bacteriostatic antibiotics that prevent the formation of dihydrofolic acid (Sköld, 2001; Masters et al., 2003; Drillia et al., 2005), which leads to the reduction of tetrahydrofolic acid, a cofactor for nucleotide synthesis (Michelow and McCracken, 2009). It constitutes up to 21% of the antibiotic drugs used in humans (Göbel et al., 2005). Approximately 15% of the administered SMX remains unmetabolized and, hence, excreted unchanged from the body (Hirsch et al., 1999; Perez et al., 2005). In urban and hospital wastewaters, the concentration of SMX has been observed up to 66.4 μ/L; in Germany, SMX concentrations between 30 and 85 ng/L have been measured in surface waters (Hartig et al., 1999). SMX is among the most detected sulfonamide antibiotics in wastewater (Göbel et al., 2007; Choi et al., 2008; Le-Minh et al., 2010), and through cation exchange or bridging, it can bind to organic matter in the soil (Xu et al., 2011).

The sulfonamide resistance gene (*sul*) is coded on the dihydropteroate synthetase (DHPS) gene (Sköld, 2000) and is spread by mobile genetic elements (MGEs) (Huovinen, 2001; Antunes et al., 2007). Four different sulfonamide resistance genes, namely, *sulA*, *sul*I, *sul*II, and *sul*III, have been defined in environmental bacteria. *sul*A is chromosomally coded on DHPS in *Streptococcus pneumoniae*, which was mutated by insertion, resulting in sulfonamide resistance (Maskell et al., 1997). *sul*I and *sul*II were detected in polluted sea and river samples (Lin and Biyela, 2005; Hu et al., 2008; Mohapatra et al., 2008), in cattle farm stool samples (Srinivasan et al., 2005), and in samples from wetland sediments (Agersø and Petersen, 2007; Akinbowale et al., 2007). *sul*I, being coded on class 1 integrons, can be transferred between bacteria in the water matrix (Tennstedt et al., 2003; Mukherjee and Chakraborty, 2006; Taviani et al., 2008).

In addition to resistance against SMX, Xu et al. (2011) found that SMX-resistant *Bacillus cereus* and *Bacillus firmus* also have the capacity to degrade SMX at high rates. Moreover, *Thauera* sp., bacteria known to have the ability to degrade aromatic compounds, were also assumed to metabolize SMX since they were abundantly detected under chronic exposure to SMX (Miran et al., 2018).

Yan et al. (2022) investigated the SMX biodegradation pathways and the fate of antibiotic-resistant genes (ARGs) in heterotrophic and autotrophic microorganisms by employing three different experimental setups: aerobic sludge, nitrifying sludge, and mixed sludge. They exposed the sludge to SMX in the range of 0.2–10 mg/L for 91 days and observed the removal of SMX in all experimental setups. Through sequencing and quantitative PCR (qPCR) analyses, they identified the microbial structure, quantified the *sul*I and *sul*II genes, and reported potential antibiotic-resistant bacteria (ARB) and SMX-utilizing bacteria after SMX exposure. The authors found that ammonia-oxidizing bacteria (AOB) could remove SMX quite efficiently, while the previously overlooked heterotrophic bacteria, although 15 times slower than the AOB, also displayed an important role in SMX removal in aerobic and mixed sludge reactors.

Cetecioglu et al. (2016) investigated the impact of SMX on the function of anaerobic processes. They loaded the system with up to 40 mg/L SMX, which was removed by biodegradation. It was found that 45 mg/L SMX was lethal for the anaerobic system. A shift in the microbial community under SMX pressure was also observed.

Müller et al. (2013) worked with a bench-scale activated sludge system, where they confirmed that the removal of SMX via adsorption was negligible. They also confirmed that SMX can be readily utilized as the energy, carbon, and/or nitrogen source for growth, presumably by the two groups of bacteria found in activated sludge communities: heterotrophic bacteria assimilating SMX as a carbon and/or nitrogen source and autotrophic nitrifying bacteria oxidizing the functional amino group on the aromatic ring of SMX. Furthermore, Müller et al. (2013) mentioned that the removal of SMX was enhanced under nitrogen-poor conditions, which is in line with the findings of Drillia et al. (2005). Based on their results, Drillia et al. (2005) concluded that SMX could be removed in systems with low readily biodegradable substrates, such as extended aeration systems.

Zhang et al. (2020) studied the removal of SMX in a long-term sulfur-based autotrophic denitrification reactor. Their results showed that SMX was removed by biodegradation and, at a concentration of 20 mg/L, did not have an impact on the nitrate removal performance of the reactor. However, SMX had a significant impact on the microbial community composition, which resulted in a shift in the autotrophic denitrifying microorganisms.

In current and previous studies (Orhon et al., 2009; Orhon and Sözen, 2012; Pala-Ozkok et al., 2014b), respirometry and the oxygen uptake rate (OUR) profiles have been used as essential tools to determine the kinetic impact of persistent pollutants on activated sludge systems with high precision. The chronic impact of other important antibiotics such as tetracycline and erythromycin was studied in the previous phase of the study on similar sludge systems at different sludge ages using respirometry as the main technique, which indicated that biodegradation, storage, and the hydrolysis kinetics were influenced by continuous antibiotic feeding. The impact of antibiotic exposure on the microbial community structure and the link between community structure and microbial kinetics have been successfully established (Pala-Ozkok and Orhon, 2013; Pala-Ozkok et al., 2019). Furthermore, Katipoglu-Yazan et al. (2016, 2018) investigated the impact of chronic exposure to SMX for 41 days on an enriched nitrifying microbial culture and showed that the nitratation and nitritation processes were significantly disturbed and the microbial community structure changed. In these previous works, respirometry was used to determine the substrate biodegradation and inhibition characteristics reflected in the OUR profiles without and with SMX. The kinetics were estimated and used for the interpretation of the short-/long-term influence of the antibiotic, and the kinetic impacts were identified on the enriched microbial cultures at the end of chronic exposure.

Existing studies on the influence of antibiotics on wastewater treatment systems have pointed out the importance of applied acclimation strategies, operational conditions, and microbial culture composition on the removal of substrates and the fate of pollutants. Studies that investigated the impact of exposure to various SMX concentrations underlined that the SMX utilization ability could be developed in activated sludge microbial populations (Drillia et al., 2005; Yan et al., 2022). Improvements in the treatment efficiencies with intermittent feeding of carbon sources were reported, particularly in anaerobic digestion for different types of substrates and wastewaters (Nadais et al., 2005; Bonk et al., 2018; Vikromvarasiri et al., 2023). However, the impact of the feeding regime, such as intermittent exposure to antibiotics, has yet to be studied on activated sludge systems.

The aim of this study was to explore the chronic impact of SMX feed, along with a peptone mixture, on a mixed microbial culture for a period of 30 days. The system was further operated without the addition of SMX followed by exposure to SMX 20 days later. In this respect, this study evaluated the acute and chronic impacts of SMX on the kinetics and the microbial community structure, as well as the SMX resistance genes (i.e., *sul*I, *sul*II, and *sul*III), in a laboratory-scale activated sludge system. Moreover, for the first time, the kinetic effect of intermittent exposure to SMX on the same system was investigated. The OUR profiles obtained via respirometry were used for the simulation and calibration of the multicomponent models consisting of the related processes and parameters for the estimation of the process/inhibition kinetics, while high-throughput 454-pyrosequencing was used for the identification of the changes in the microbial community.

## 2 Materials and methods

### 2.1 Reactor operation and experimental setup

The control reactor was a 14-L laboratory-scale fill/draw reactor that was established using the seed sludge taken from the aeration tank of a full-scale domestic wastewater treatment plant in Istanbul. The hydraulic (*θ*_H_) and sludge retention time (SRT, *θ*_X_), i.e., sludge age, were 1 and 10 days, respectively. The dissolved oxygen (DO) concentration in the reactor was maintained above 3.0 mg/L at all times to ensure aerobic conditions. The reactor was acclimated to a peptone–meat extract mixture (peptone mixture), a recognized standard substrate for respirometric inhibition studies (Insel et al., 2006) that includes different fully biodegradable chemical oxygen demand (COD) fractions resembling real domestic wastewater. The peptone mixture per 1 L solution consisted of 16 g peptone [pancreatic digest of gelatin (Peptone G) 7182A], 3 g urea (Urea Agar Base 7226), 11 g beef extract (Beef Extract Powder 7228A; all from Acumedia, Lansing, MI, USA), 0.4 g CaCl_2_.2H_2_O, 0.7 g NaCl, 2.8 g K_2_HPO_4_, and 0.2 g MgSO_4_.7H_2_O. The reactor feed solution contained macro- and micronutrient solutions in addition to the peptone mixture, which consisted of K_2_HPO_4_, KH_2_PO_4_, FeSO_4_.7H_2_O, MgSO_4_.7H_2_O, MnSO_4_.H_2_O, ZnSO_4_.7H_2_O, and CaCl_2_.2H_2_O. For each addition of 1,000 mg COD peptone mixture, 20 mL from both solutions were added to the reactor. The control reactor was fed 600 mg COD/L of the peptone mixture every day, which reached a steady-state biomass concentration of 2,000 mg VSS/L.

In addition to the acute experiments, the control reactor also served as the source of the seed sludge for the chronic reactor, which was also fully aerated and operated at the same sludge age of 10 days. The concentration of the peptone mixture added to the reactor was adjusted to 720 mg COD/L for the chronic inhibition tests. In addition to the peptone mixture, the feed also included a daily dosing of 50 mg SMX/L (CAS no. 723-46-6) for 30 days. Furthermore, the chronic reactor was covered to avoid photodegradation of the antibiotic compound (Kümmerer, 2009).

The dose of 50 mg/L SMX utilized in this study was chosen as a representation of concentrated streams, such as pharmaceutical plants and hospitals (Chelliapan et al., 2006; Larsson et al., 2007). Moreover, previous studies by the group showed that, when applying the ISO 8192 method (ISO, 2007) at 50 mg/L concentration, the OUR value decreased from 90 to 57 mg O_2_/L.h after 30 min of exposure (Ozkok et al., 2011). **Table 1** outlines the characteristics of the experimental runs.

**Table 1:** Characteristics of the experimental runs.

Runs		Antibiotic conc. (mg/L)	Peptone COD (mg/L)	Antibiotic COD (mg/L)	Total COD (mg/L)
Run 1	Control	–	600	0	600
Run 2	Acute	50	600	70	670
Run 3	Chr day 24	Only population and SMX analysis			
Run 4	Chr day 30	50	720	70	790
Run 5	Intermittent day 50	50	720	70	790

COD, chemical oxygen demand; Chr, chronic; SMX, sulfamethoxazole.

### 2.2 Analytical procedures

Polyhydroxyalkanoate (PHA) samples were collected and analyzed according to Beun et al. (2000). Volatile suspended solids (VSS) and suspended solids (SS) were measured according to standard methods (APHA, 2012). Firstly, soluble COD samples were filtered through 0.45-μm Millipore membrane filters and the measurements then conducted according to the ISO 6060 procedure (ISO, 1989).

**Figure 1:**
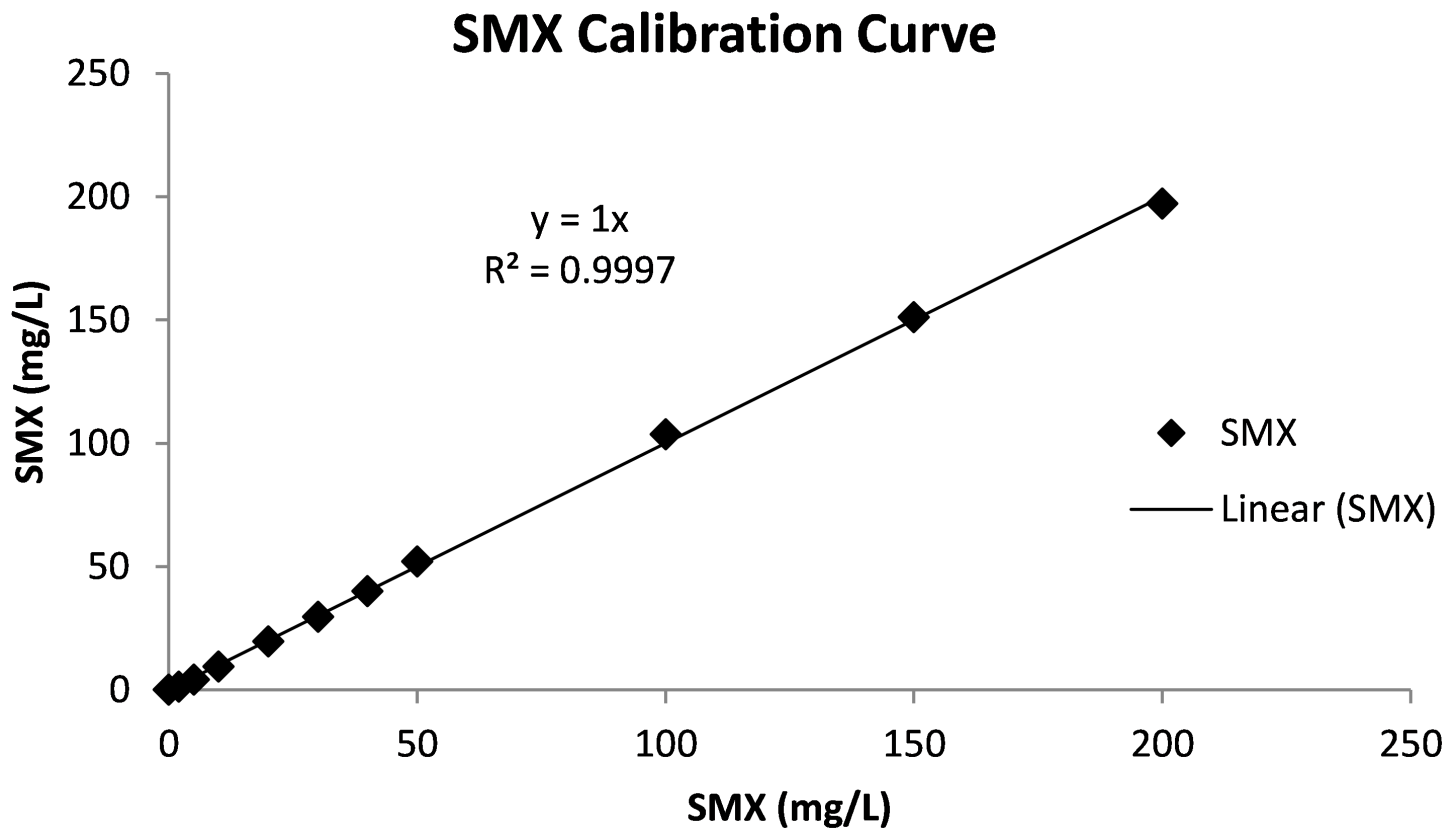
Calibration curve of sulfamethoxazole. Reproduced from Pala-Ozkok et al. (2012).

SMX measurements were conducted using an Agilent high-performance liquid chromatography (HPLC) equipped with a Nova-PaK C18 column (Waters, Milford, MA, USA) and a diode array detector at 280 nm. The mobile phase had a constant flow rate at 0.6 mL/min and consisted of 30:70 (*v*/*v*) methanol/water, which was acidified (pH 2.5) using 0.1% phosphoric acid (Beltran et al., 2008). The sample injection volume and the flow rate were 40 µL and 1 mL/min, respectively. The calibration curves for the SMX measurements are given in **Figure 1**.

### 2.3 Respirometric analysis and activated sludge modeling

The Ra-COMBO (Applitek Co., Nazareth, Belgium) continuous respirometer was used to produce the OUR profiles in all experimental runs. For this purpose, parallel reactors were set up to have identical conditions for all runs. To prevent any interference due to nitrification, a nitrification inhibitor (Formula 2533™; Hach, Loveland, CO, USA) was added into the OUR reactors during all analyses. The OUR tests were started and ended at endogenous decay level, at which no external carbon source was present. The respirometric reactor was continuously aerated, and the COD and polyhydroxybutyric acid (PHB) samples were collected in all experimental sets.

The model used in this study for the kinetic interpretation of the experimental data included the basic structure of the activated sludge model (ASM) for growth and storage (Gujer et al., 1999; Krishna and Van Loosdrecht, 1999). A matrix representation of the structure of the model is given in **Table 2**. Moreover, the AQUASIM simulation program was used for the modeling and simulation of the OUR data obtained from the different experimental runs (Reichert, 1994), where all model outputs were fitted on real-time data by manual calibration of the model components.

**Table 2:** Matrix representation of the model structure for simultaneous growth and storage.

Component→ Process↓	*S*_O_	*S*_S_	*S*_H1_	*S*_H2_	*X*_H_	*X*_P_	*S*_P_	*X*_STO_	Rate equations
Growth of *X*_H_	$-\frac{1-Y_{\text{H}}}{Y_{\text{H}}}$	$-\frac{1}{Y_{\text{H}}}$			1				${\hat{\mu }}_{H}\left(\frac{S_{S}}{K_{S}+S_{S}}\right)\left(\frac{S_{O}}{K_{OH}+S_{O}}\right)X_{H}$
Hydrolysis of *S*_H1_		1	−1						$k_{h1}\left(\frac{S_{H1}/X_{H}}{K_{X}+S_{H1}/X_{H}}\right)\left(\frac{S_{O}}{K_{HH}+S_{O}}\right)X_{H}$
Hydrolysis of *S*_H2_		1		−1					$k_{h2}\left(\frac{S_{H2}/X_{H}}{K_{XX}+S_{H2}/X_{H}}\right)\left(\frac{S_{O}}{K_{OH}+S_{O}}\right)X_{H}$
Storage of *X*_STO_	$-\left(1-Y_{\text{STO}}\right)$	−1						*Y*_STO_	$k_{STO}\left(\frac{S_{S}}{K_{STO}+S_{S}}\right)\left(\frac{S_{O}}{K_{OH}+S_{O}}\right)X_{H}$
Growth on *X*_STO_	$-\frac{1-Y_{\text{H}}}{Y_{\text{H}}}$				1			$-\frac{1}{Y_{\text{H}}}$	${\hat{\mu }}_{STO}X_{STO}\left(\frac{S_{O}}{K_{OH}+S_{O}}\right)$
Decay of *X*_H_	$-\left(1-f_{\text{E}}\right)$				−1	*f*_EX_	*f*_ES_		$b_{H}X_{H}\left(\frac{S_{O}}{K_{OH}+S_{O}}\right)$
Parameters	O_2_	COD	COD	COD	Cell COD	COD	COD	COD	

COD, chemical oxygen demand.

Full description of parameters is available in **Table 4**.

### 2.4 Activated sludge bacterial community and resistance gene analyses

In order to determine the impact of chronic exposure to SMX on the microbial community structure, DNA samples were taken from the chronic reactor on day 0 (run 1), day 24 (run 3), and day 30 (run 4) and analyzed using 454-pyrosequencing. Details of the methodology for community analysis have been given in several previous publications of the group (Pala-Ozkok et al., 2012, 2014a, 2019). Genomic DNA was extracted from the activated sludge samples using the NucleoSpin Soil DNA extraction kit (Macherey-Nagel GmbH&Co, Düren, Germany). The 27F and 338R barcoded universal primers were used to amplify the V1–V2 hypervariable regions of the 16S rRNA gene. The amplified DNA from each sample was pooled in one sample and sent to the Institute for Clinical Molecular Biology at the University of Kiel for sequencing (Pala-Ozkok et al., 2014a). PANGEA PERL scripts were used to screen the obtained sequences for quality and length (Giongo et al., 2010). MOTHUR was used as the main tool for statistical and operational taxonomic unit (OTU)-based analyses. Moreover, significant changes at the species level were determined using the metastats command in MOTHUR, with a *p*-value threshold of 0.05 (Schloss et al., 2009). After the cleanup, the numbers of sequences obtained in each sample were 2,977, 3,118, and 2,752 for run 1, run 3, and run 4, respectively. The sequence reads were uploaded to the Sequence Read Archive (SRA) and made available under accession number SRA054103. The accession numbers for run 1, run 3, and run 4 are SRX155362/155363, SRX155377, and SRX155378, respectively. Moreover, the SMX resistance profile was analyzed using PCR of the *sul*I, *sul*II, and *sul*III genes in the activated sludge samples according to the method described in Pei et al. (2006) and Pala-Ozkok (2012). The primer sequences for *sul*I, *sul*II, and *sul*III and the annealing temperatures are given in **Supplementary Table S1**.

## 3 Results and discussions

### 3.1 Assessment of the oxygen uptake rate profiles and SMX utilization

**Figure 2:**
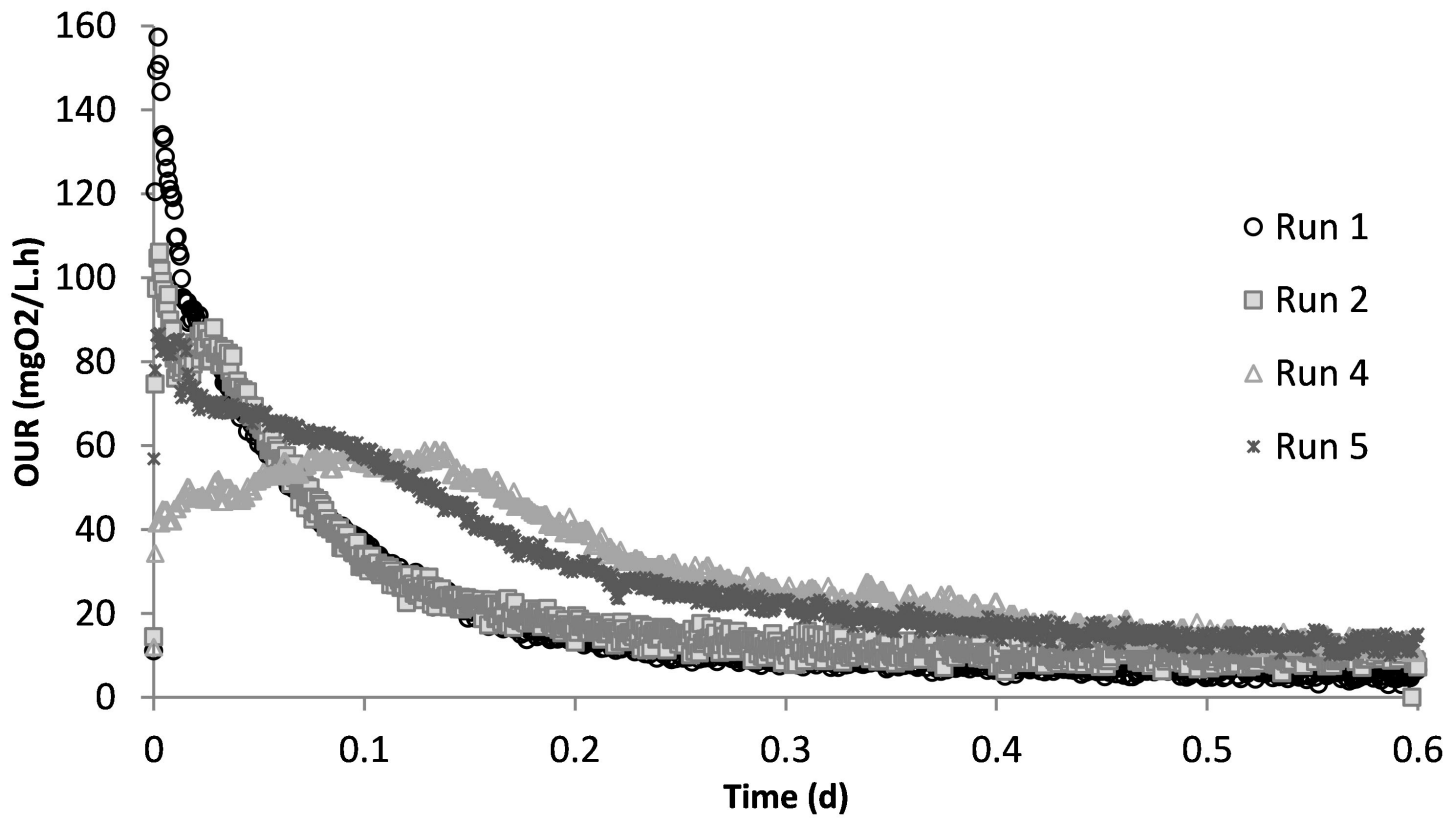
Oxygen uptake rate (OUR) profiles obtained from different experimental runs.

Run 1 served as the control test, where there was no impact of SMX on the biomass. **Figure 2** displays the OUR curve for the peptone mixture biodegradation, which showed the peak of the curve at 160 mg/L.h and later dropping to the endogenous decay line. The curve presented the degradation of different COD fractions, of which the peptone mixture was included, with the corresponding degradation rates. The total oxygen consumption calculated from the area under the OUR curve and the corresponding COD removal efficiency were 211 mg/L and 94%, respectively (Pala-Ozkok et al., 2012; Pala-Ozkok and Orhon, 2013; Pala-Ozkok et al., 2019).

The acute impact of the addition of 50 mg/L SMX on the biomass from the control reactor was visualized using the obtained OUR profile (**Figure 2**). SMX addition caused the peak to drop from 160 mg/L.h in run 1 to 106 mg/L.h in run 2, and the oxygen consumed for microorganism growth in run 2 was calculated as 206 mg/L. In the chronic run (run 4), the total amount of oxygen consumed for growth was 278 mg/L. On day 50 (run 5), after 20 days of no SMX addition, the oxygen consumption was calculated as 275 mg/L.

**Figure 3:**
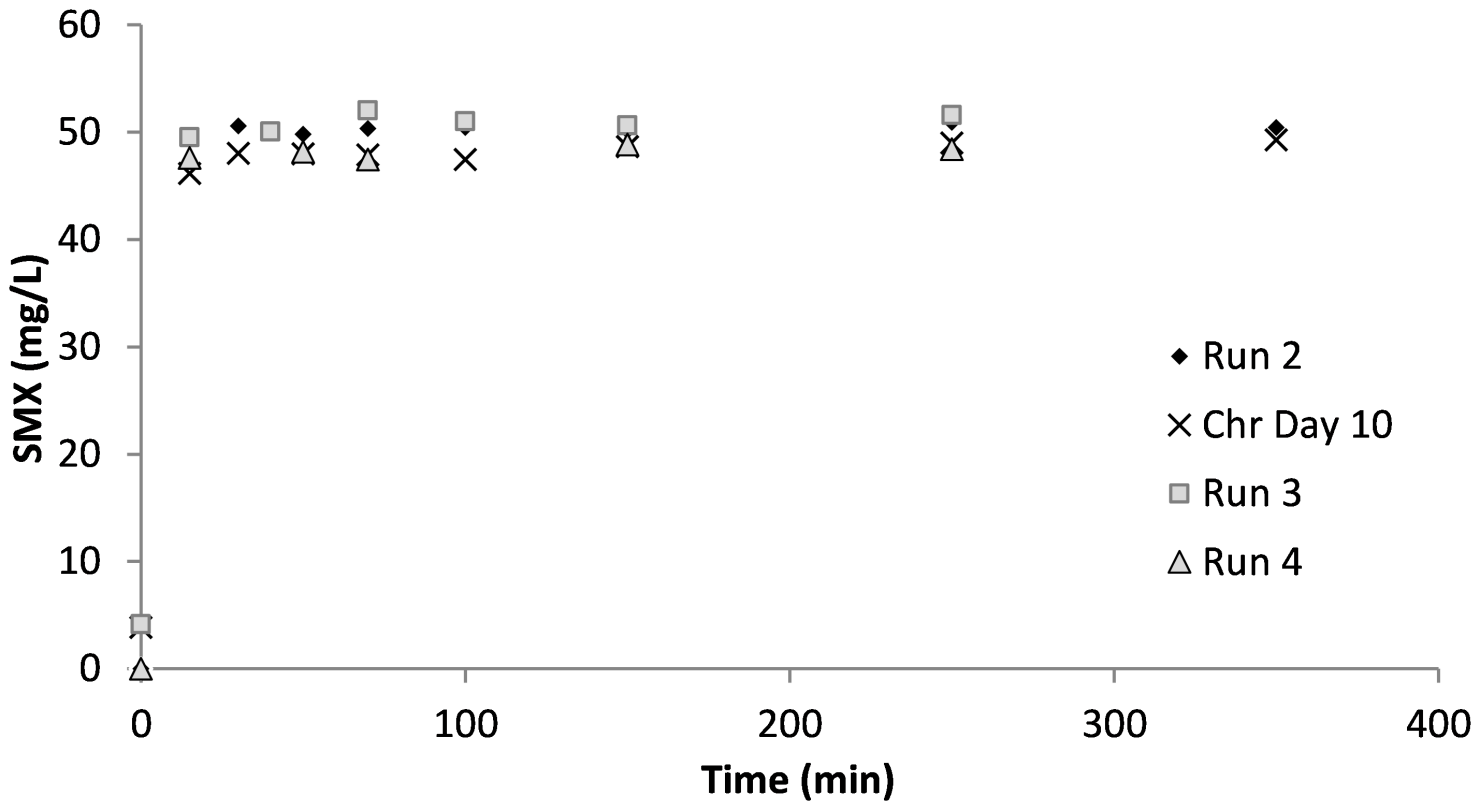
Effluent sulfamethoxazole (SMX) concentrations in the respirometric tests and in the chronic reactor.

**Figure 3** shows the SMX concentrations measured in the samples taken during the acute and the chronic experimental runs. Additional measurements were performed on days 10 and 24 of chronic exposure. As can be seen, all of the SMX was measured in the liquid phase, which is in agreement with the literature indicating that SMX is not adsorbed onto the sludge (Yan et al., 2022). However, contrary to the findings by Yan et al. (2022), the results of the SMX measurements in this study showed that the biomass did not utilize SMX as a carbon or nitrogen source. Since all of the SMX was measured in the liquid phase, it was concluded that there was no contribution to oxygen consumption in the respirometric tests.

### 3.2 Substrate binding impact of sulfamethoxazole

In previous works of the group, the substrate-binding feature of antibiotic substances has been explained and reported in detail for SMX, as well as for erythromycin and tetracycline, under different operating conditions, i.e., with and without nitrification, with different substrates, and at different sludge ages (Ozkok et al., 2011; Pala-Ozkok et al., 2014, 2014b; Kor-Bicakci et al., 2016; Katipoglu-Yazan et al., 2013, 2016, 2018, 2023). In all experimental runs, the OUR profiles were started from the endogenous decay line, in which there was no external substrate present in the medium. At the end of the experiment, the OUR profiles reached the endogenous decay line, indicating that all of the external biodegradable substrate was depleted. Therefore, the decrease in the amount of oxygen utilized during the experiments, i.e., the area under the OUR curve, indicated the utilization of less substrate by the biomass.

The data from the experimental results, including those obtained from previous studies by the group conducted on the same origin of activated sludge on different antibiotics (Katipoglu-Yazan et al., 2013; Pala-Ozkok and Orhon, 2013; Pala-Ozkok et al., 2014; Katipoglu-Yazan et al., 2016; Kor-Bicakci et al., 2016; Pala-Ozkok et al., 2019), suggest that the antibiotic compounds “blocked” a portion of the peptone mixture added to the reactor and prevented its utilization in the microbial growth process, resulting in less oxygen consumption and low effluent COD. **Table 3** presents the mass balance created for the experimental sets. For the mass balance calculations, the yield for heterotrophic growth (*Y*_H_) and that for the residual soluble microbial product, *S*_P_, (*Y*_SP_) were determined from the control experiment (run 1) as 0.6 mg cell COD/mg COD and 0.06 mg COD/mg COD, respectively. These values were used for the rest of the experimental runs. In run 2, the COD initially available was 600 mg/L; however, the mass balance calculations showed that 85 mg COD/L (14%) of this initially available amount was not utilized for growth. In run 4 and run 5, the amounts of “blocked” COD, which was not used for growth, were calculated as 25 mg COD/L (3.6%) and 33 mg COD/L (4.6%), respectively.

**Table 3:** Oxygen consumption and chemical oxygen demand (COD) utilization mass balances in the experimental runs.

Run	SMX conc. (mg/L)	Initial peptone mixture COD (mg/L)	Oxygen consumed (mg/L)	COD utilized (mg/L)	COD bound (mg/L)	Remaining soluble COD (mg/L)		
						Total	Soluble metabolic product, *S*_P_	Peptone mixture + SMX
Run 1	–	600	211	600	–	36	36	–
Run 2	50	600	206	515	85	182	31	151
Run 4	50	720	278	695	25	122	42	80
Run 5	50	720	275	688	33	135	41	94

SMX, sulfamethoxazole.

Examination of the impact of other antibiotics in the studies conducted by the group (Pala-Ozkok and Orhon, 2013; Pala-Ozkok et al., 2014b; Katipoglu-Yazan et al., 2013; Pala-Ozkok et al., 2019) revealed different substrate binding impacts of acute and chronic exposures and differences in the type of antibiotics. For a sludge age of 10 days, where nitrification was inhibited, acute exposure to tetracycline resulted in 29% of the COD not being utilized for growth, while chronic exposure resulted in 30% of the COD not utilized. For erythromycin, the acute impact resulted in 52%, chronic exposure resulted in 18%, and intermittent exposure resulted in 25% of the COD not being used for growth. Compared with erythromycin and tetracycline, SMX showed definitely weaker substrate binding properties; nonetheless, it had an impact, and this impact was also seen in the kinetics of activated sludge.

Moreover, extensive work by the group showed that the type of carbon source, which the system was acclimated to, and the culture history (i.e., sludge age), together with the operational conditions (i.e., presence of nitrification), changed the outcome of the SMX impact (Katipoglu-Yazan et al., 2016, 2018). The acute impact of SMX was also shown to differ depending on the type of substrate (Pala-Ozkok et al., 2014), wherein, on an acetate-acclimated sludge at a sludge age of 2 days, SMX only exerted a slight inhibition on microbial growth, with full substrate utilization. In addition, the acute impact on a 10-day SRT system acclimated to acetate showed mild substrate binding, while the chronic impact showed SMX used as a secondary carbon source (Kor-Bicakci et al., 2016). Moreover, in another part of the study where nitrification was not prevented, it was seen that, after 50 mg/L acute SMX exposure, only half of the initial peptone COD was utilized, with the rest of the COD reported as “blocked” (Katipoglu-Yazan et al., 2023).

As a result of this evaluation, the substrate binding feature of antibiotics was taken into consideration. Moreover, the mass balances presented in **Table 3** were created and used as the input data for activated sludge modeling studies.

### 3.3 Model evaluation of the impact of sulfamethoxazole

**Figure 4:**
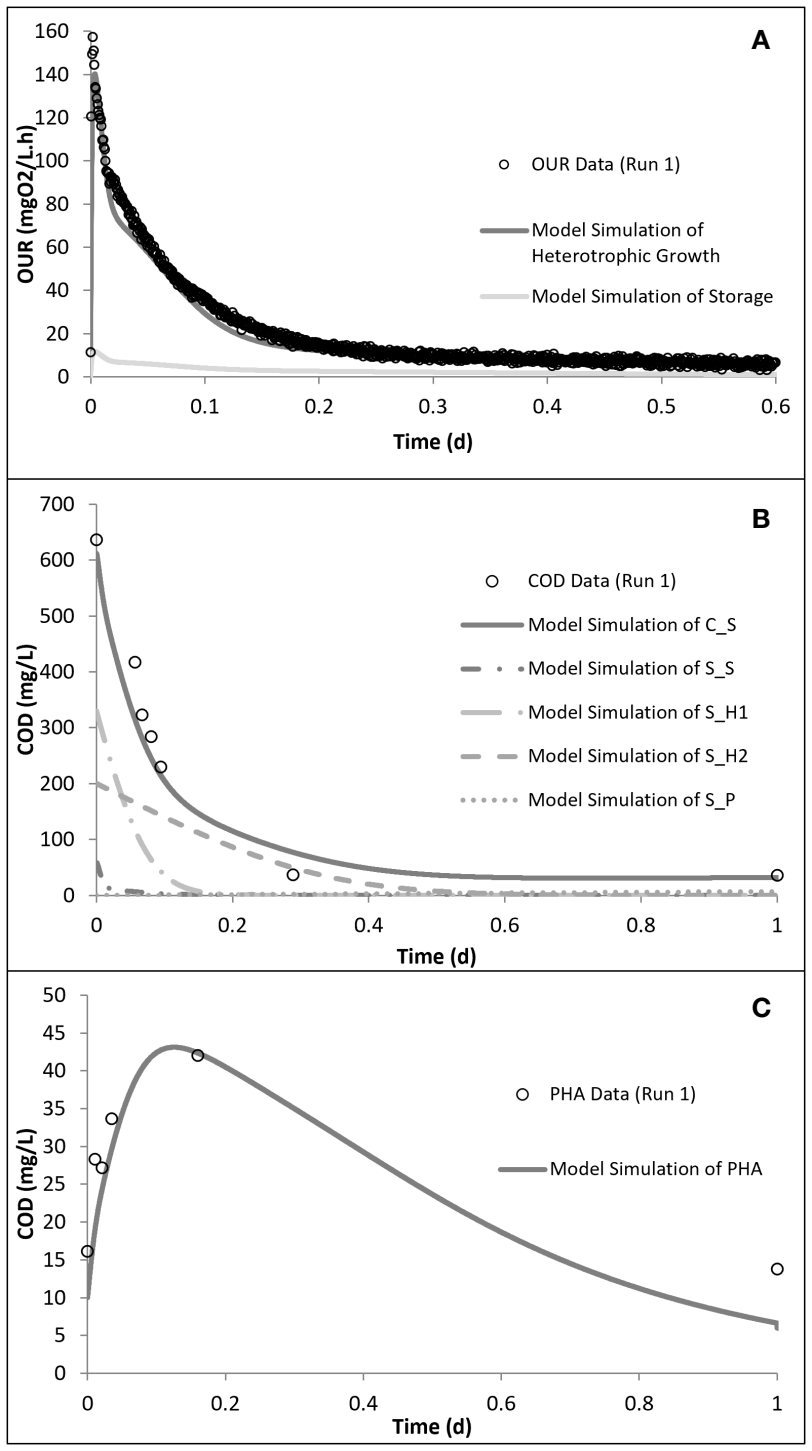
Model simulation of run 1: oxygen uptake rate (OUR) **(A)**, chemical oxygen demand (COD) removal **(B)**, and polyhydroxyalkanoate (PHA) storage **(C)** profiles. Reproduced from Pala-Ozkok et al. (2012, 2019).

The peptone mixture consisted of three different COD fractions: the readily biodegradable (*S*_S_), readily hydrolysable (*S*_H1_), and hydrolysable (*S*_H2_) COD fractions. The model calibration of run 1 showed that the readily biodegradable and readily hydrolysable fractions constituted 9.5% and 56%, while the hydrolysable COD fraction constituted 34.5% of the total biodegradable COD added into the reactor. PHA analysis in run 1 showed that the control system had a PHA pool of 10 mg COD/L and a maximum PHA storage of 32 mg COD/L. However, in the acute and chronic experimental runs, the results of the PHA measurements showed that SMX inhibited the storage mechanism completely, which was also observed for tetracycline and erythromycin (Katipoglu-Yazan et al., 2013; Pala-Ozkok and Orhon, 2013; Pala-Ozkok et al., 2019). The OUR, COD, and PHA profiles and the model simulations of run 1 are given in **Figure 4**.

**Figure 5:**
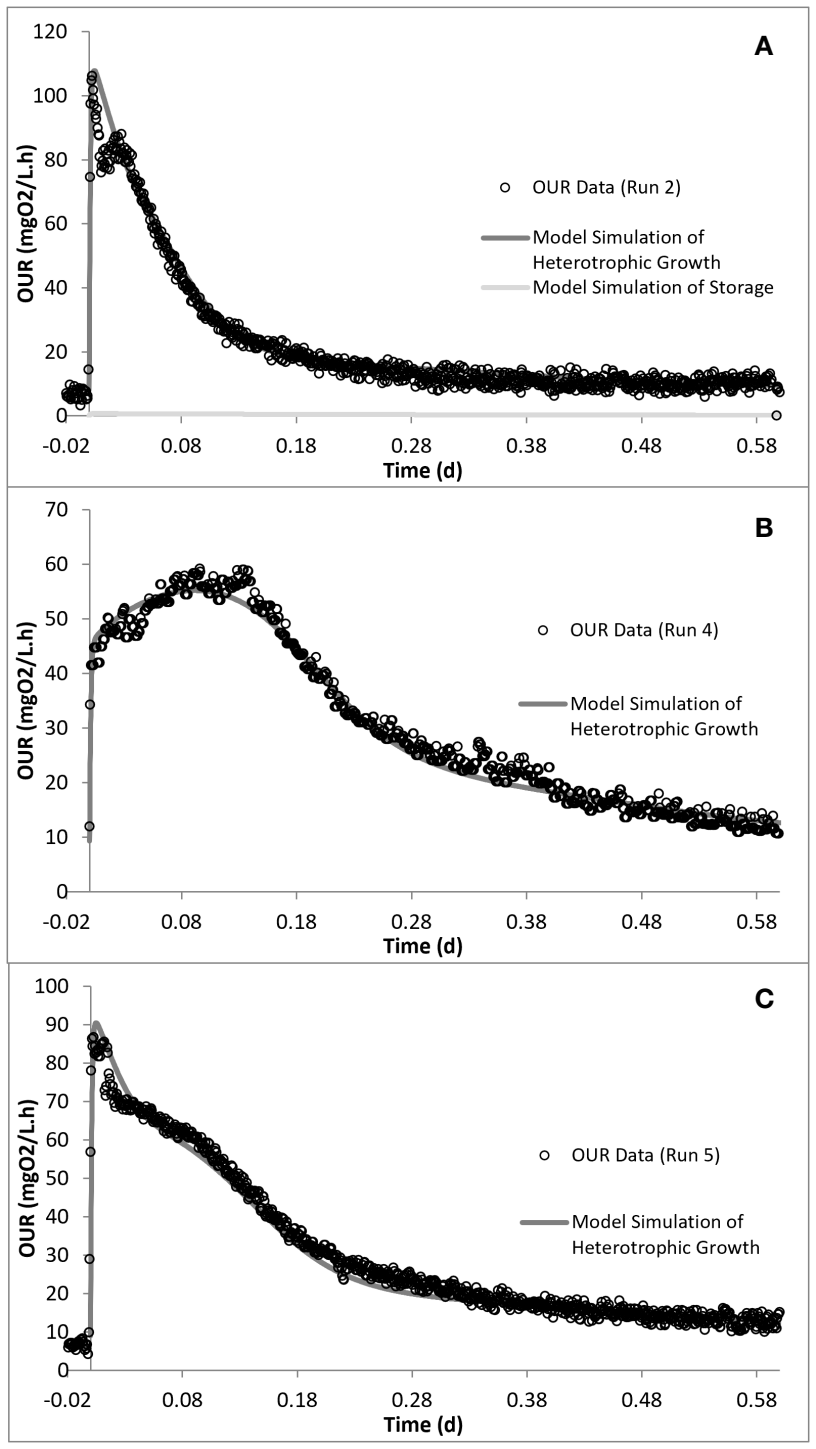
Model simulation of the oxygen uptake rate (OUR) profiles for run 2 **(A)**, run 4 **(B)**, and run 5 **(C)**.

**Figure 6:**
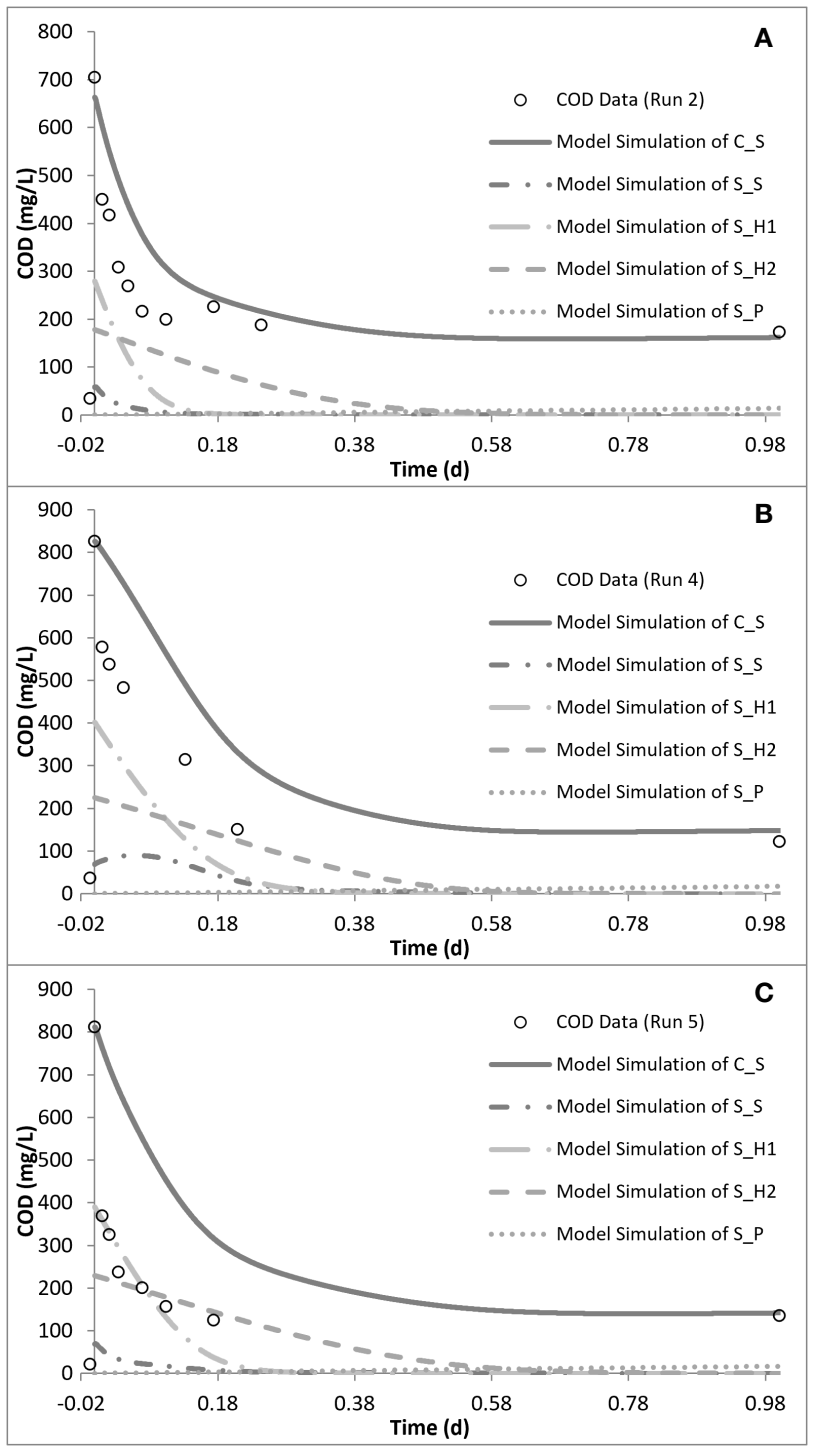
Model simulation of the chemical oxygen demand (COD) removal profiles for run 2 **(A)**, run 4 **(B)**, and run 5 **(C)**.

Kinetic analysis of the impact of acute inhibition (run 2) on the storage mechanism showed that, although the PHA storage was inhibited, the biomass maintained the ability to grow on the stored PHA. In addition, it was shown that the addition of SMX increased the half-saturation constant (*K*_S_), making it less available for the growth of biomass (**Table 4**). It has also been determined that the biomass did not utilize all of the COD added into the reactor, but 85 mg COD/L less than the amount added for run 2. Furthermore, the addition of SMX doubled the endogenous decay level (*b*_H_) of the system (**Figures 5**, **6**).

**Table 4:** Model calibration of the peptone mixture removal kinetics with sulfamethoxazole (SMX) addition.

Model parameter		Unit	Run 1	Run 2	Run 4	Run 5
Maximum growth rate for *X*_H_	${\hat{\mu }}_{\text{H}}$	1/day	5.2	5.2	3	5.2
Half-saturation constant for growth of *X*_H_	*K*_S_	mg COD/L	24	40	80	50
Endogenous decay rate for *X*_H_	*b*_H_	1/day	0.1	0.2	0.27	0.27
Heterotrophic half-saturation coefficient for oxygen	*K*_OH_	mg O_2_/L	0.01	0.01	0.01	0.01
Maximum hydrolysis rate for *S*_H1_	*k*_h_	1/day	5.2	5.2	3.9	3.8
Hydrolysis half-saturation constant for *S*_H1_	*K*_X_	g COD/g COD	0.15	0.15	0.21	0.15
Maximum hydrolysis rate for *X*_S1_	*k*_hx_	1/day	0.56	0.56	0.56	0.56
Hydrolysis half-saturation constant for *X*_S1_	*K*_XX_	g COD/g COD	0.05	0.05	0.05	0.05
Maximum storage rate of PHA by *X*_H_	*k*_STO_	1/day	1.2	0	0	0
Maximum growth rate on PHA for *X*_H_	${\hat{\mu }}_{STO}$	1/day	0.8	0.8	0	0
Half-saturation constant for PHA storage by *X*_H_	*K*_STO_	mg COD/L	0.5	0.5	0	0
Yield coefficient of *X*_H_	*Y*_H_	g COD/g COD	0.6	0.6	0.6	0.6
Yield coefficient of PHA	*Y*_STO_	g COD/g COD	0.8	0.8	0.8	0.8
Fraction of biomass converted to *S*_P_	*f*_ES_	–	0.05	0.05	0.05	0.05
Fraction of biomass converted to *X*_P_	*f*_EX_	–	0.15	0.15	0.15	0.15
State variables						
Total biomass		mg COD/L	2,010	1,891	1,640	1,846
Initial active biomass	*X*_H1_	mg COD/L	1,450	1,200	932	1,000
Activity		%	72	64	57	54
Initial amount of PHA	*X*_STO1_	mg COD/L	10	16	0	0
Initial amount of biodegradable COD	*C*_S1_	mg COD/L	600	600	720	720
Initial amount of readily biodegradable COD	*S*_S1_	mg COD/L	57	57	68	68
Initial amount of readily hydrolysable COD	*S*_H1_	mg COD/L	335	280	402	390
Initial amount of hydrolysable COD	*S*_H2_	mg COD/L	208	178	225	229
Bound COD		mgCOD/L	–	85	25	33

PHA, polyhydroxyalkanoate; COD, chemical oxygen demand.

Simulation of the chronic exposure data (run 4) showed that exposure to 50 mg/L SMX for 30 days resulted in the increase of the half-saturation constant (*K*_S_) of the substrate from 24 mg COD/L in run 1 to 80 mg COD/L in run 4. Moreover, the maximum growth rate of the microorganisms decreased from 5.1 day^−1^ in run 1 to 3 day^−1^ in run 4, impacting both the growth and substrate degradation (**Table 4**). Moreover, chronic exposure to SMX for 30 days almost tripled the endogenous decay level, which increased from 0.1 day^−1^ in run 1 to 0.27 day^−1^ in run 4. The rate of hydrolysis (*k*_h1_) of *S*_H1_ decreased from 5.2 day^−1^ in run 1 to 3.9 day^−1^ in run 4, while the half-saturation constant for the hydrolysis (*K*_X_) of *S*_H1_ increased by 1.4-fold in run 4 compared with run 1. Finally, consistent with the stoichiometric calculations presented in **Table 3**, the model simulation showed that the system utilized 25 mg COD/L less than the amount provided (**Figures 5**, **6**).

In order to observe the effects of intermittent exposure to antibiotics, after 30 days of exposure to 50 mg/L SMX, the addition of SMX was stopped for 20 days and the reactor was fed only with the peptone mixture. On day 50, 50 mg/L SMX was again added to the system, with the responses obtained and simulated in run 5 (**Figures 5**, **6**). The hydrolysis rate of *S*_H1_ remained the same when compared with that in run 4, while the half-saturation constant for *S*_H1_ hydrolysis recovered to its original level in run 1. The maximum heterotrophic growth rate, ${\hat{\mu }}_{\text{H}}$ also recovered from 3 day^−1^ in run 4 to 5.2 day^−1^ in run 5, as it was in run 1. A recovery in the half-saturation constant (*K*_S_) for the growth of *X*_H_ was also observed. Finally, the endogenous decay rate (*b*_H_) of the biomass remained at 0.27 day^−1^, as in day 30 (run 4), almost tripling the endogenous decay rate of the organisms. These results indicated some recovery in the 20 days of intermittence as the impact was not as severe as that on the 30th day (run 4). However, 33 mg COD/L of the amount provided was not utilized by the system. Finally, it was observed that, in both acute and chronic exposure runs, the hydrolysis of *S*_H2_ remained unaffected (**Table 4**).

### 3.4 Microbial community analysis

**Figure 7:**
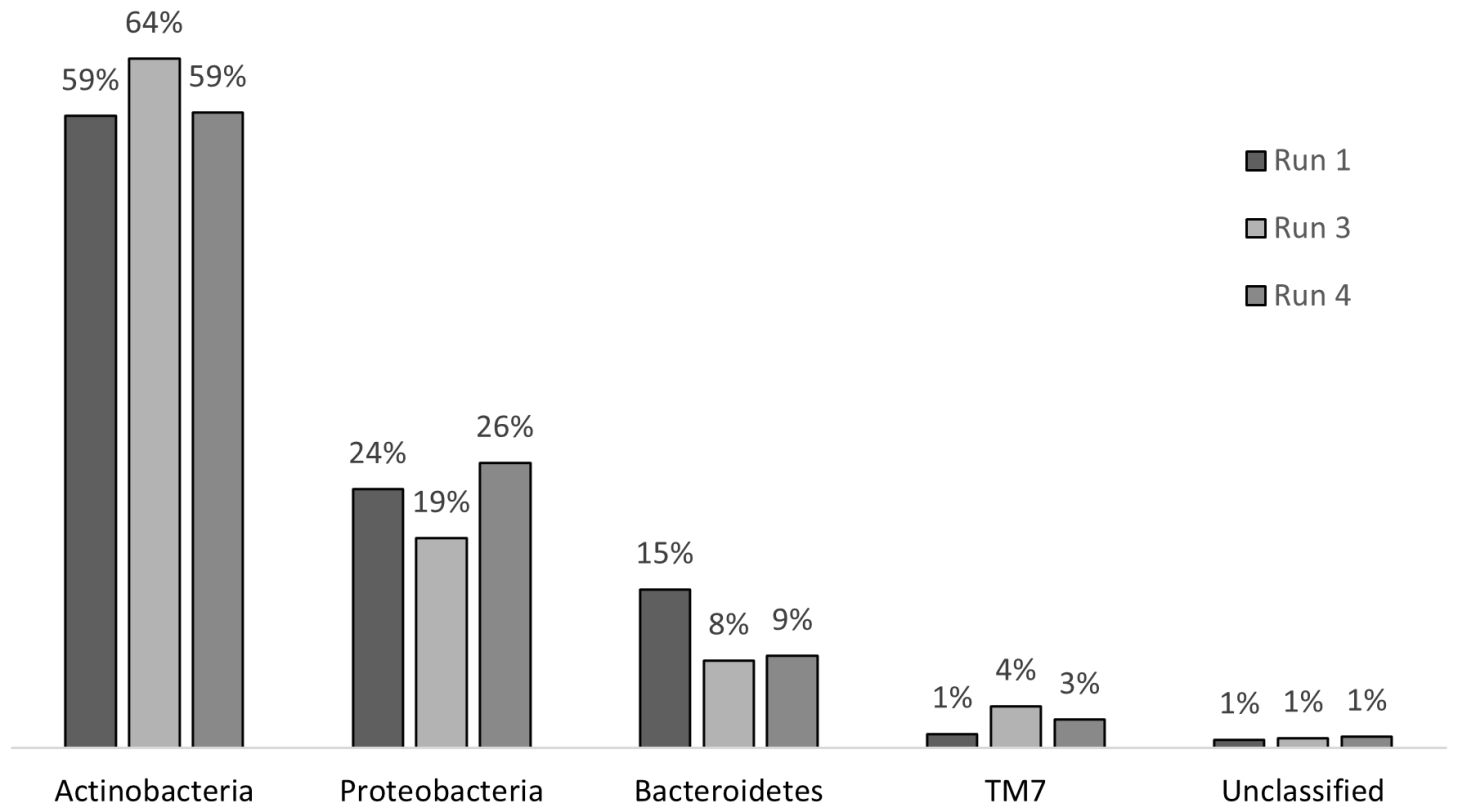
Bacterial community structures at the phylum level for run 1, run 3, and run 4.

Chronic exposure to SMX caused stress on the microbial community; however, this stress did not result in a shift in the community structure at the phylum level (**Figure 7**). In run 1, where there was no impact of SMX, the phylum structure of the community consisted of 59% Actinobacteria, 15% Bacteroidetes, 24% Proteobacteria, and 1% TM7. After 24 days of exposure in run 3, the abundance rates changed to 64%, 8%, 19%, and 4% for Actinobacteria, Bacteroidetes, Proteobacteria, and TM7, respectively. In run 4, there was a change back to the original structure with regard to the abundance of Actinobacteria and Proteobacteria. However, the abundance of the phyla Bacteroidetes and TM7 remained the same as that in run 3. The change in the distribution of phyla is presented in **Figure 7**. The changes in Actinobacteria and Proteobacteria between run 1 and run 3 were also determined to be significant when compared with the RDP library, which also showed that, throughout the treatment, the decrease in the abundance of Bacteroidetes was significant. However, as a result of the pressure of SMX, Proteobacteria, Actinobacteria, and Bacteroidetes continued to dominate the community as the most abundant phyla in the reactor, which is in accordance with the findings of Zhang et al. (2016).

**Figure 8:**
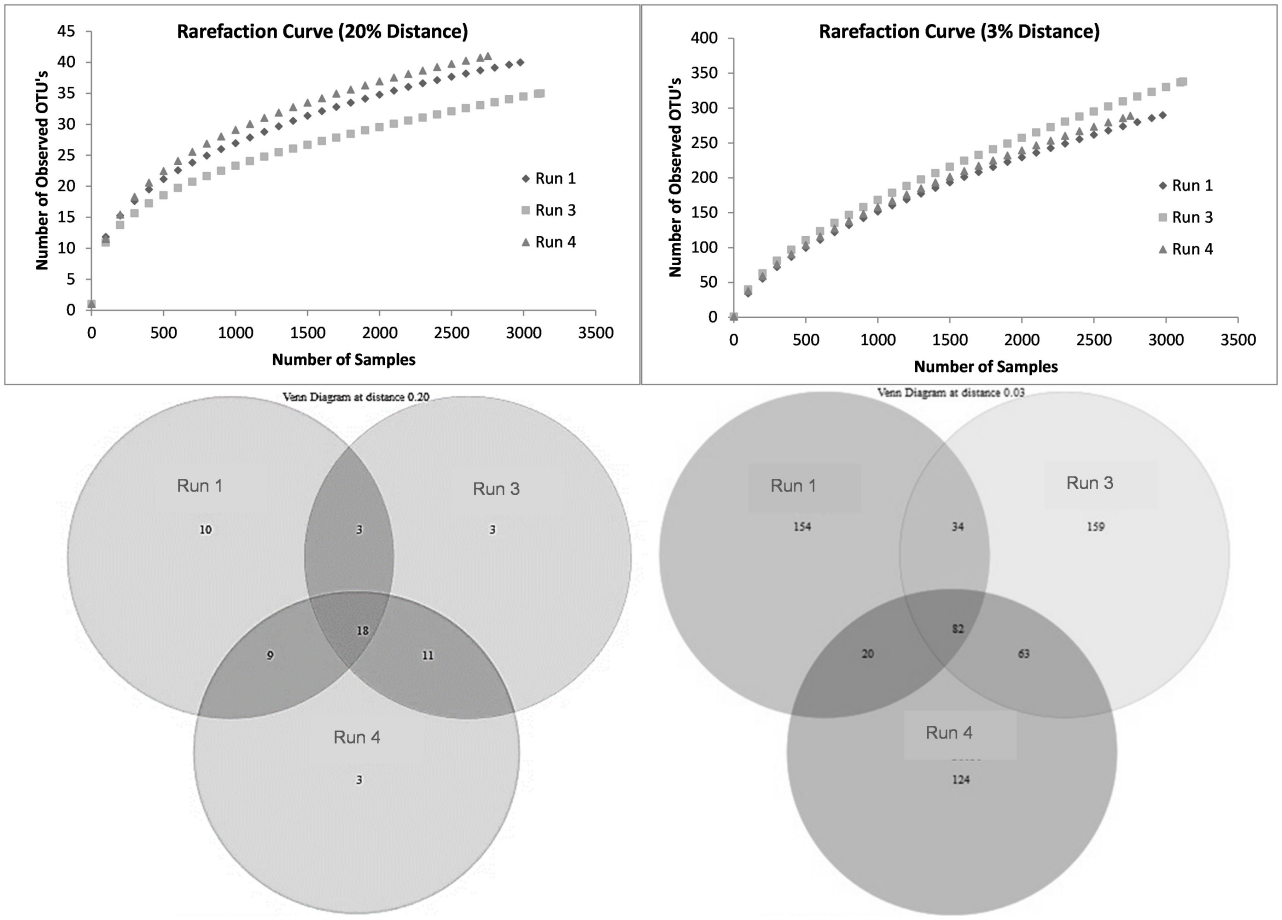
Rarefaction curves and Venn diagrams at the phylum (20%) and species (3%) levels for run 1, run 3, and run 4.

The change in richness due to SMX exposure was visualized using rarefaction curves. At the species level, the richness showed an increasing trend, with run 3 having the highest richness (**Figure 8**). According to the data in the Venn diagrams, the total richness of all groups combined was calculated to consist of 636 species-level OTUs (**Figure 8**), while the total shared richness was calculated as consisting of 82 species-level OTUs. At the species level, run 1, run 3, and run 4 contained 288, 338, and 289 species-level OTUs, respectively. Run 1 and run 3 exclusively shared 34 species-level OTUs, while run 1 and run 3 exclusively shared 20 and 63 species-level OTUs, respectively. Moreover, the ACE and Chao1 richness estimators indicated that the richness increased by the 24th day of exposure (run 3); however, this slightly decreased back to the level of run 1 by the 30th day (run 4) (**Table 5**). Assessment of the Shannon diversity index (*H*) revealed that, at the species level, the diversity increased with the addition of SMX into the system (**Table 5**). An increase in the Shannon diversity index was also observed in the study of Zhang et al. (2016), where activated sludge was exposed to 10 mg/L SMX.

**Table 5:** Statistical indicators for sulfamethoxazole (SMX) exposure.

	3%			20%		
	Run 1	Run 3	Run 4	Run 1	Run 3	Run 4
No. of OTUs	288	338	289	42	35	41
Singleton	168	206	169	14	14	13
Chao1 richness estimator	647.7	807.2	619.1	55.0	65.3	52.1
ACE richness estimator	1,019.5	1,278.6	1,058.1	66.9	78.2	56.4
Shannon diversity index (*H*)	3.0	3.6	3.6	1.6	1.5	1.6
Evenness	0.53	0.62	0.63	0.41	0.41	0.43
Good’s coverage (%)	41.67	39.05	41.52	66.67	60.00	68.29

OTUs, operational taxonomic units.

**Table 6** outlines the significant changes in the community structure at the species level. It can be seen that an unclassified member of class Actinobacteria (OTU#6) and an unclassified member of class Chitinophagaceae (OTU#10) were the most abundant species in run 1, with 45% and 10% abundance, respectively. However, in run 3 (after 24 days of SMX exposure), OTU#10 vanished and did not reappear (*p*< 0.05, *q* > 0.05). On the other hand, OTU#6 gradually decreased from 45% down to 9% in run 4 (30 days of exposure). Moreover, bacteria that were in very low abundance in run 1 increased significantly and dominated the community. Species of the genus *Arthrobacter* OTU#2 and OUT#55 became gradually abundant, reaching 24% and 10% in run 4 (30 days of exposure), with total abundance of 34% for this genus. In addition, an unclassified member of class Chitinophagaceae (OTU#340) showed increased abundance in 30 days and reached 6% abundance in run 4.

**Table 6:** Significant changes in the activated sludge population under sulfamethoxazole (SMX) impact.

Phylum	Nearest classified neighbor	OTU number	Run 1 (%)	Run 3 (%)	Run 4 (%)
Actinobacteria	*Arthrobacter*	2	0	2	24
	*Arthrobacter*	55	1	9	10
	Unclassified Intrasporangiaceae	6	45	28	9
	Unclassified Actinomycetales	18	1	7	4
Bacteroidetes	Unclassified Chitinophagaceae	10	10	0	0
	Unclassified Chitinophagaceae	340	0	6	6
Proteobacteria	*Paracoccus*	3	1	2	3
	Unclassified Sphingomonadaceae	7	3	0	0
	Unclassified Comamonadaceae	19	4	1	1
	Unclassified Comamonadaceae	24	0	2	3

OTU, operational taxonomic unit.

### 3.5 Evaluation of changes in the microbial community

Comparison of the results of the pyrosequencing analysis with those in the available literature indicated that the dominant bacteria in this system were resistant to SMX, which was also cross-checked with the results of the resistance gene analysis of the system. The results showed that the system harbored two sulfonamide resistance genes, namely, *sul*I and *sul*II, but not *sul*III. *sul*I and *sul*II were detected in all samples in run 1, run 3, and run 4, whereas *sul*III was not found in any of the samples (**Supplementary Table S2**). It was also shown that these sulfonamide resistance genes, particularly *sul*I, are located on mobile genetic elements, and the class 1 integron has been known to harbor *sul*I (Liebert et al., 1999; Carattoli, 2001; Byrne-Bailey et al., 2009; Baran et al., 2011). Zhang et al. (2016) showed that, under tetracycline and SMX pressure, the total number of the antibiotic resistance genes they investigated (i.e., *tet*A, *tet*C, *tet*G, *tet*K, *tet*M, and *sul*I) increased. Furthermore, the authors revealed that the change in the antibiotic resistance genes was significantly correlated with the change in the class 1 integron. Moreover, Hoa et al. (2008) showed that *Arthrobacter* sp., found abundantly in the SMX reactor, was positive for all three *sul* genes. A positive correlation with multidrug resistance genes was also found for Chitinophagaceae (Shen et al., 2019). In addition, members of the family Intrasporangiaceae were found to assimilate SMX in soil, as discovered by Ouyang et al. (2019). Since SMX was not degraded in this study, it is safe to assume that the members of Intrasporangiaceae found in this study are resistant to SMX. Yan et al. (2022) determined the functional bacteria potentially carrying the *sul* resistance genes in their systems under chronic exposure to SMX. The authors found, among others, *Comamonas* and *Taibaiella* species as two of the potential ARB, which is consistent with the results of this study as these species belong to the Comamonadaceae and Chitinophagaceae families, respectively, the members of which were also identified in this study (**Table 6**), investigated the *sul* resistance genes.

## 4 Conclusions

This study investigated the acute and chronic impacts of the addition of SMX on an activated sludge system. The impact of intermittent exposure was also explored for the first time. These impacts were evaluated from different points of view: microbial kinetics, antibiotic biodegradation, microbial community structure, and antibiotic resistance. The results indicated that SMX was not used as a carbon or nitrogen source by the biomass, but had a mild impact on the substrate degradation kinetics of the activated sludge biomass, which included an increase in the half-saturation constant for heterotrophic growth (*K*_S_) and the endogenous decay level (*b*_H_). This impact was more pronounced in the chronic exposure studies. However, when compared with previous studies conducted using erythromycin and tetracycline, the impact is at a lower level, with SMX also having an impact on substrate binding. All the kinetic and stoichiometric effects of SMX on the biomass decreased after 20 days of intermittence. The microbial community structure was not severely affected by SMX; however, potentially resistant species were identified, where a mild community shift at the species level occurred.

## Data availability statement

The datasets presented in this study can be found in online repositories. The names of the repository/repositories and accession number(s) can be found in the article/**Supplementary Material**.

## Author contributions

IP-O: Investigation, Methodology, Writing – original draft, Writing – review & editing. TK-Y: Investigation, Writing – review & editing. TO-H: Methodology, Writing – review & editing. DJ: Supervision, Writing – review & editing. EU-C: Supervision, Writing – review & editing. DO: Funding acquisition, Supervision, Writing – review & editing.
