# Supplementary material for: Impact of acute and chronic exposure to sulfamethoxazole on the kinetics and microbial structure of an activated sludge community
Source: Front Antibiot. 2024 Apr 2;3:1335654. doi: 10.3389/frabi.2024.1335654 (PMC11732045; doi:10.3389/frabi.2024.1335654)
Supplement: Supplementary file 1 [file Table_1.docx]

**Supplamentary Table 1.** Primers used for the determination of sulfonamide resistance genes.

| Gene | Primers | Sequence | Annealing Temperature | Amplicon Size | Reference |
| --- | --- | --- | --- | --- | --- |
| *sul*I | *sul*I-FW | cgcaccggaaacatcgctgcac | 55.9 | 163 | (Pei et al*.*, 2006) |
|  | *sul*I-RV | tgaagttccgccgcaaggctcg |  |  |  |
| *sul*II | *sul*II-FW | tccggtggaggccggtatctgg | 60.8 | 191 |  |
|  | *sul*II-RV | cgggaatgccatctgccttgag |  |  |  |
| *sul*III | *sul*III-FW | tccgttcagcgaattggtgcag | 60.0 | 128 |  |
|  | *sul*III-RV | ttcgttcacgccttacaccagc |  |  |  |
