# Supplementary material for: Impact of acute and chronic exposure to sulfamethoxazole on the kinetics and microbial structure of an activated sludge community
Source: Front Antibiot. 2024 Apr 2;3:1335654. doi: 10.3389/frabi.2024.1335654 (PMC11732045; doi:10.3389/frabi.2024.1335654)
Supplement: Supplementary file 2 [file Table_2.docx]

Supplementary Table 2. Results of sulfanomide resistance gene analysis

| Sample | *sul*I | *sul*II | *sul*III |
| --- | --- | --- | --- |
| Positive Control | + | + | - |
| Run 1 | + | + | - |
| Run 3 | + | + | - |
| Run 4 | + | + | - |
| NTC | - | - | - |
